# Experimental Air Warming of a *Stylosanthes capitata*, Vogel Dominated Tropical Pasture Affects Soil Respiration and Nitrogen Dynamics

**DOI:** 10.3389/fpls.2017.00046

**Published:** 2017-02-01

**Authors:** Miquel A. Gonzalez-Meler, Lais B. C. Silva, Eduardo Dias-De-Oliveira, Charles E. Flower, Carlos A. Martinez

**Affiliations:** ^1^Ecology and Evolution, Department of Biological Sciences, University of Illinois at ChicagoChicago, IL, USA; ^2^Department of Biology, University of São PauloRibeirao Preto, Brazil

**Keywords:** nitrogen, stable isotopes, warming, soil respiration, temperature, tropic, pasture, productivity

## Abstract

Warming due to global climate change is predicted to reach 2°C in tropical latitudes. There is an alarming paucity of information regarding the effects of air temperature on tropical agroecosystems, including foraging pastures. Here, we investigated the effects of a 2°C increase in air temperature over ambient for 30 days on an established tropical pasture (Ribeirão Preto, São Paulo, Brazil) dominated by the legume *Stylosanthes capitata* Vogel, using a T-FACE (temperature free-air controlled enhancement) system. We tested the effects of air warming on soil properties [carbon (C), nitrogen (N), and their stable isotopic levels (δ^13^C and δ^15^N), as well as soil respiration and soil enzymatic activity] and aboveground characteristics (foliar C, N, δ^13^C, δ^15^N, leaf area index, and aboveground biomass) under field conditions. Results show that experimental air warming moderately increased soil respiration rates compared to ambient temperature. Soil respiration was positively correlated with soil temperature and moisture during mid-day (when soil respiration was at its highest) but not at dusk. Foliar δ^13^C were not different between control and elevated temperature treatments, indicating that plants grown in warmed plots did not show the obvious signs of water stress often seen in warming experiments. The ^15^N isotopic composition of leaves from plants grown at elevated temperature was lower than in ambient plants, suggesting perhaps a higher proportion of N-fixation contributing to tissue N in warmed plants when compared to ambient ones. Soil microbial enzymatic activity decreased in response to the air warming treatment, suggesting a slower decomposition of organic matter under elevated air temperature conditions. Decreased soil enzyme capacity and increases in soil respiration and plant biomass in plots exposed to high temperature suggest that increased root activity may have caused the increase seen in soil respiration in this tropical pasture. This response along with rapid changes in soil and plant ^15^N may differ from what has been shown in temperate grasslands.

## Introduction

Air temperature is a major abiotic factor governing the CO_2_ flux both to and from terrestrial ecosystems worldwide (Davidson and Janssens, 2006; Taneva and Gonzalez-Meler, 2011; Hopkins et al., 2013). Temperature is expected to increase worldwide as a result of climate change (Lu et al., 2013), with the potential to increase ecosystem carbon losses over photosynthetic C gains (Gonzalez-Meler et al., 2004; Ciais et al., 2005; De Lucia et al., 2007). Consequently, changes in the productivity of ecosystems may exacerbate the rate at which C accumulates in the atmosphere (Valentini et al., 2000; Luo et al., 2001). Additional evidence suggests the length and frequency of warm spells have increased in the second half of the twentieth century (IPCC, 2013). Extreme or episodic weather events, such as heat waves or late season warming combined with different patterns of precipitation (including drought events) may further climatic effects on plants and ecosystems (Schimel et al., 2001; Ciais et al., 2005; Dukes et al., 2005; Cerri et al., 2007; Krause et al., 2013). Recent experimental warming studies have shown that increased temperature may increase leaf-level and canopy photosynthesis, with inconsistent results on aboveground NPP and soil respiration (Lin et al., 2010; Hopkins et al., 2013; Lu et al., 2013; Gonzalez-Meler et al., 2014). However, these studies have taken place in temperate or northern ecosystems with a myriad of results (Hopkins et al., 2013). Tropical regions and tropical ecosystems are not represented in warming studies (Schimel et al., 2001; Wood et al., 2012; Gonzalez-Meler et al., 2014), with information desperately needed because the tropics play a particularly important role in the terrestrial carbon budget (Raich et al., 2006). In the tropics, climate and land use change have reduced tree cover in favor of agriculture (Anadón et al., 2014). Tropical rangelands represent the single most dominant land use type in Brazil, but little is known about their vulnerabilities to sustained or episodic weather events, such as increases in air temperature (Webb et al., 2011). This is particularly true for belowground processes, such as soil respiration, microbial enzymatic capacity or N dynamics, as the existing warming studies are inconclusive regarding belowground responses to changes in air temperature (Lin et al., 1999; Hopkins et al., 2013).

Soil respiration is the largest ecosystem flux after gross primary photosynthesis (Gonzalez-Meler et al., 2004) and it is largely influenced by changes in photosynthetic C uptake, temperature and precipitation (Davidson et al., 2000). Total soil respiration is composed of respiration of roots and that of soil heterotrophs, which may differ in temperature sensitivities (Bahn et al., 2009; Taneva and Gonzalez-Meler, 2011). Long-term soil warming experiments have shown the expected initial increases in soil respiration followed by a long-term decrease or no change (Melillo et al., 2002; Tang et al., 2006). In grasslands, experimental and seasonal warming has led to confounding effects of soil respiration responses to photosynthesis, soil moisture and temperature (Luo et al., 2001; Bahn et al., 2009; Gomez-Casanovas et al., 2012). Global warming and episodic heat wave events during the growing season will likely lead to increases in soil respiration, evapotranspiration and changes in plant growth (Davidson et al., 2000; Petheram et al., 2012). Enzymatic activities from soil heterotrophic microorganisms may also be affected by increases in air temperature (Zhao et al., 2013).

Some enzymes like dehydrogenase, β-glucosidase and urease are important when studying soil decomposition processes. Soil dehydrogenases are an enzyme responsible for catalyzing the oxidation of organic substrates, indicating the oxidative soil capacity. As such, dehydrogenase is a proxy for the overall microbial activity but it is not proportional with the microbial biomass present in the soil (Burns and Dick, 2002; Figueiredo et al., 2010). β-glucosidase drives the catalytic hydrolysis of cellulose and is positively correlated with the content of plant-derived soil organic matter (Burns and Dick, 2002; Figueiredo et al., 2010). More directly associated with N cycling, urease catalyzes the hydrolysis of urea molecules, producing ammonia and CO_2_. High urease activity produces ammonia, whilst low urease activity reduces ammonia production and N availability for plants (Burns and Dick, 2002).

Here we report the effects of a 30-day warming event (+2°C over ambient air temperature) on a tropical pasture located in Brazil within the Trop-T-FACE (Tropical Temperature Free Air Concentration Enhancement) experiment. The experimental warming timeframe coincided with the normal post-graze plant regrowth practice period for the region. We measured soil respiration, soil enzymes, and plant and soil δ^13^C, and δ^15^N to understand how abiotic drivers (temperature and water content) impact a Brazilian forage crop system. Our primary objectives were to understand the relationship of soil respiration with the variation of plant biomass, soil temperature and soil moisture in a tropical pasture system. We hypothesized that soil respiration and enzymatic activity will be stimulated by the +2°C air warming treatment. We further hypothesized that the increases in soil respiration in response to experimental air warming will be caused by increases in plant activity and not by passive responses of respiration to soil moisture or temperature.

## Materials and methods

### Site description and Trop-T-FACE design and system performance

The field experiment was conducted at the campus of the University of São Paulo (21° 10′ 08.4″ S and 47° 51′ 50.6″ W; elevation 578 m) in the municipality of Ribeirão Preto, São Paulo State, in March 2013. Climate of the region is classified as subtropical humid (Cwa or Cfa) (Peel et al., 2007), with hot and rainy summers (30°C; 200 mm of rain/month; 80% relative humidity) and cold and dry winters (13°C; 30 mm rain/month; 60% relative humidity). Soils at the site are classified as dystrophic red latosols, representing roughly 23% of all tropical soils. Soils are deep, well drained, and uniform throughout the profile with firm, low granular structure and low pH. Soils was fertilized with N-P-K 8-30-8 (1 ton/ha) and pH was corrected with calcareous rock addition (2.5 ton/ha) to reach a uniform pH field value of 5.0.

Rangelands in the neotropics utilize legume species, such as *S. capitata* and others grown in consortium with C_4_ grasses. However, we established a 2500 m^2^ plantation with *S. capitata* only, a native C_3_ leguminous forage species widely used in tropical pastures. There were 16 experimental 11 × 11 m plots (8 control plots and 8 heated plots) containing a 2 m diameter experimental temperature manipulation ring.

*Stylosanthes capitata* was planted from seed. After establishment, plants grew to a height of 60 cm (about 40 days) without irrigation. Plants were then cut to a height of 10 cm to stimulate grazing. Plants were then grown to a height of 60 cm for a second time for 30 days under the temperature treatments.

**Figure 1:**
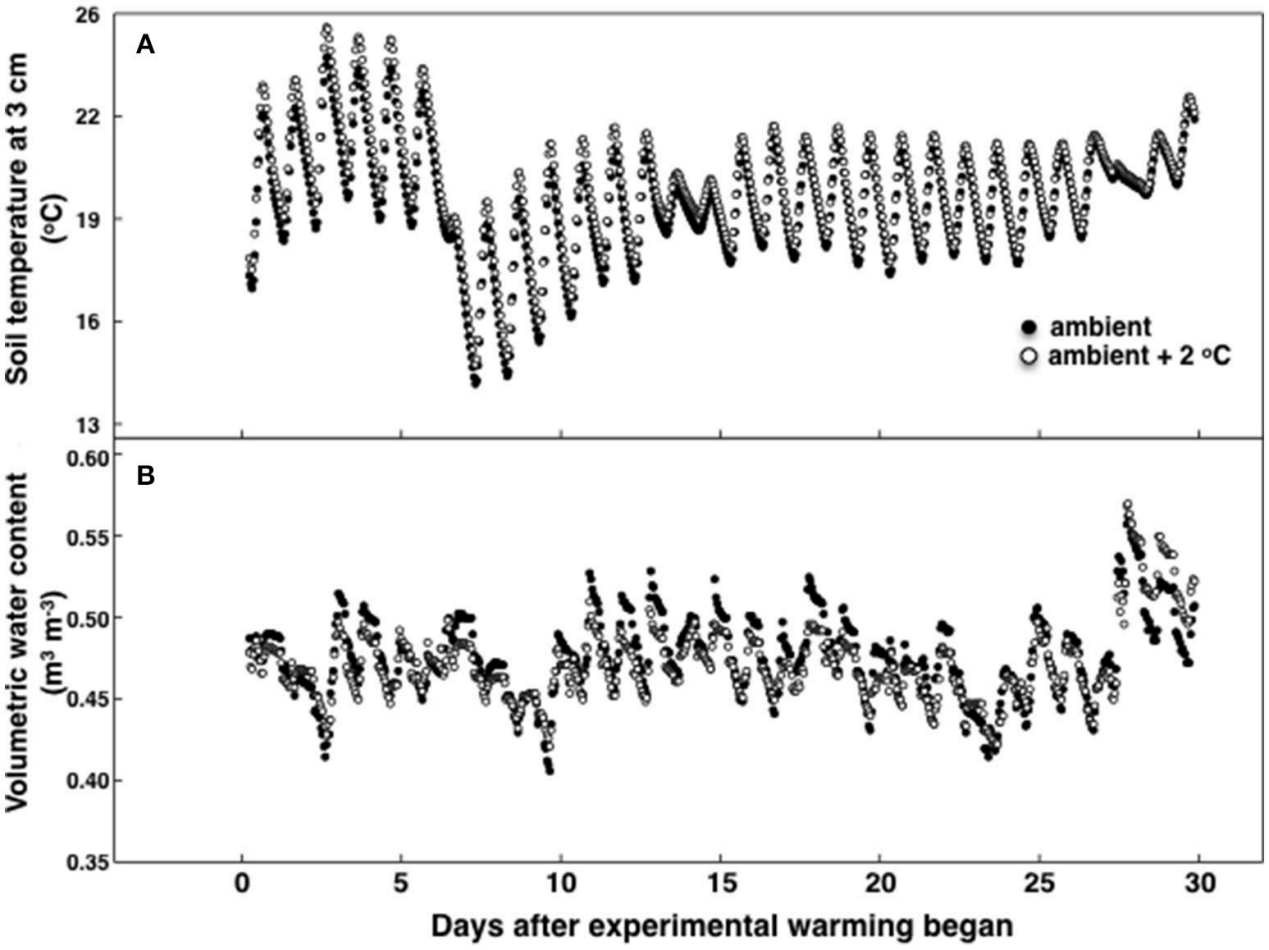
**Soil temperature (A)** and soil moisture **(B)** during the 30-day period of the temperature treatment measured in either 8 ambient temperature plots or 8 ambient +2°C air temperature plots. Standard errors are not shown for simplicity and were no more than 9.2% of the mean for temperature and 6.2% for moisture.

The Trop-T-FACE system was developed following the T-FACE system described elsewhere (Kimball, 2005; Kimball et al., 2007). In brief, the Trop-T- FACE consisted of the following components: a central control unit and data storage unit, 8 electrical control panels units for distribution and power control in the heated plots. All heated 2 m diameter plots contained 6 infrared ceramic heating elements, (model FTE-750-240, Mor Electric, USA) and target temperature was controlled by 16 infrared thermometers (model SI-1H1-L20, Apogee Instruments, USA) in heated and ambient plots. The infrared heaters were arranged in a hexagonal array on aluminum reflectors (Salamander ALEX, Mor Electric, USA). Similar arrays with dummy heaters were deployed on the ambient plots. The heaters were controlled using a PID control system (Kimball, 2005) connected to a datalogger (CR1000 with AM25T multiplexors, Campbell Scientific, USA) and monitored with LoggerNet software (Campbell Scientific, USA). The Trop-T-FACE system allowed for the maintenance of a set point of 2°C of difference in canopy temperature between ambient and heated plots (see Figure 1).

During the experimental period the highest recorded canopy temperature was 30.1 and 31.8°C in the ambient and heated plots, respectively. The lowest recorded canopy temperature was 9.0 in the ambient and 11.0°C in the heated plots. Soil temperature, measured at 10 cm deep, was 20.1°C ± 0.5 at 12:00 h and 21.1°C ± 0.3 at 18:00 h at ambient temperature (Figure 1). For the elevated temperature treatment, soil temperature at 12:00 h was 20.9°C ± 0.4 and 21.8°C ± 0.4 at 18:00 h. On average air canopy temperature was 1.9°C ± 0.2 and soil temperature was 0.8°C ± 0.1 higher under the elevated temperature treatment, than in ambient plots (Figure 1). Soil moisture was also sampled at 10 cm deep. The volumetric soil water content was lower during the temperature experimental period than during the pretreatment period, but there were no differences in between treatments (Figure 1B). Precipitation decreased during the experimental period compared to the first growth cycle without a temperature treatment (data not shown).

### Biomass and leaf area index measurements

Above ground biomass of *S. capitata* plants grown under ambient and +2°C warming was sampled after 30 days of treatments. Harvested material was oven dried at 60°C until constant weight (~48 h). The leaf area index (LAI) of the ambient and warmed plots were measured with a SunScan Canopy Analysis System (Delta-T Devices, UK).

### Soil respiration measurements

Soil respiration rates were measured with a field-portable infrared gas analyzer (IRGA; LiCor 8100-A, Lincoln, Nebraska, USA) in PVC collars, randomly placed within each plot. There were 2 collars per plot permanently inserted into the soil (5 cm deep) 1 day before treatments started, and collars were open to rainfall and litterfall, except during measurements (Taneva and Gonzalez-Meler, 2011). The chosen measurement times were 12:00 h (11:00–13:00 h) and 18:00 h for all plots (18:00–20:00 h). Measurements were carried out at the beginning and at the end of the 30-day warming period for three consecutive days. Soil temperature and moisture were continuously monitored using the sensors *Theta Probe* ML2x for moisture *Theta Probe* ST2 for temperature coupled to a DL2 datalogger (Delta-T Devices, UK).

### Soil enzymatic analysis

We collected soil samples from three different random points within each plot to a depth of 15 cm. Samples from each plot were field homogenized and immediately placed on ice until analysis in the laboratory. The processing and analysis of samples were performed at UNESP-Jaboticabal (São Paulo, Brazil). Activities of the enzymes β-glucosidase (catalysis of cellulose hydrolysis), dehydrogenase (catalysis of organic reactions) and urease (catalysis of urea) were performed according to Kandeler and Gerber (1988), Schinner et al. (2012).

Dehydrogenase activity was analyzed by the addition of the electron acceptor triphenyltetrazolium chloride (TTC) to the soil sample. The TCC is reduced to colorless trifenilformazan (TPF). The TPF product red coloring is then quantified by spectrophotometry in the visible region at 485 nm (Kandeler and Gerber, 1988). The substrate used for the evaluation of the β-glucosidase activity was p-nitrophenyl-β-glucosidase, which is colorless, but the product of the reaction is yellow, and determined by spectrophotometry at 400 nm (Schinner et al., 2012). Urease activity was measured by the indophenol colorimetric method with urea as the substrate (Caldwell, 2005) and the amount of ammonium released over 24 h was quantified at 578 nm.

### Isotopic analysis

Soil and plant material were dried at 65°C until a constant weight was obtained and then ground to a fine powder for elemental and isotope analyses. A 1–2 mg sub sample was placed in a tin capsule and then combusted in an elemental analyzer (Costech Analytical, ECS 4010) coupled with an isotope ratio mass spectrometer (ThermoFinnigan Delta Plus XL equipped with Conflo III, Gas Bench II) operating in a continuous flow mode. From these analyses, we obtained both isotope ratio (δ^13^C; δ^15^N) and elemental content (%C; %N) for carbon and nitrogen of plant tissues and soils. The C and N stable isotopic composition were expressed as a “delta” notation according to:

$$\begin{array}{l} \delta^{15}\text{N or }\delta^{13}\text{C}=\left(\text{Rsample}/\text{Rstd}-1\right)\;\times \;1000\;\left(1\right), \end{array}$$

where R is the ratio of ^13^C/^12^C or ^15^N/^14^N of the sample and standard (std). The isotopic standard for C is the Pee Bee Dolomite (PBD) and for N is the atmospheric air. Stable isotope analyses were carried out at the University of Illinois at Chicago (USA).

### Statistical analysis

A repeated measures nested factorial generalized linear mixed-effects model (GLIMMIX) with a Gaussian distribution and identity link function was used to analyze the effects of the plot, collar within plot, treatment (ambient *vs*. elevated temperature), time (1200 and 1800), and day (days after initiation of temperature treatment 28, 29, 30) on soil respiration rates. Plot and collar within plot were the random effects in the model while treatment, time, and day and all the associated interactions were the fixed model effects. To account for the correlation between days, the autoregressive order one covariance structure was used. Prior to analysis, we tested the appropriateness of including soil moisture and temperature as covariates and the results indicated that there was no significance of the variables and thus no need for inclusion in the final model. Furthermore, residuals were tested after analysis to evaluate the assumptions of normality and homogeneity of variance. Visually, the residuals looked to be normally distributed except for a few outliers. The homogeneity of variance assumption was met via the Levene's test. Data was analyzed using SAS 9.3 (SAS Institute Inc.).

Analysis of variance (ANOVA, α = 0.05) and *post-hoc* Tukey's tests (*p* < 0.05) were performed independently for soil enzymatic activity and isotopic composition. Again, data were checked for normality and homogeneity of variance prior to analysis. Data was analyzed using SAS 9.3 (SAS Institute Inc.), “MYSTAT” (Systat Software) and “R i386 2.15.3” packages.

## Results

**Figure 2:**
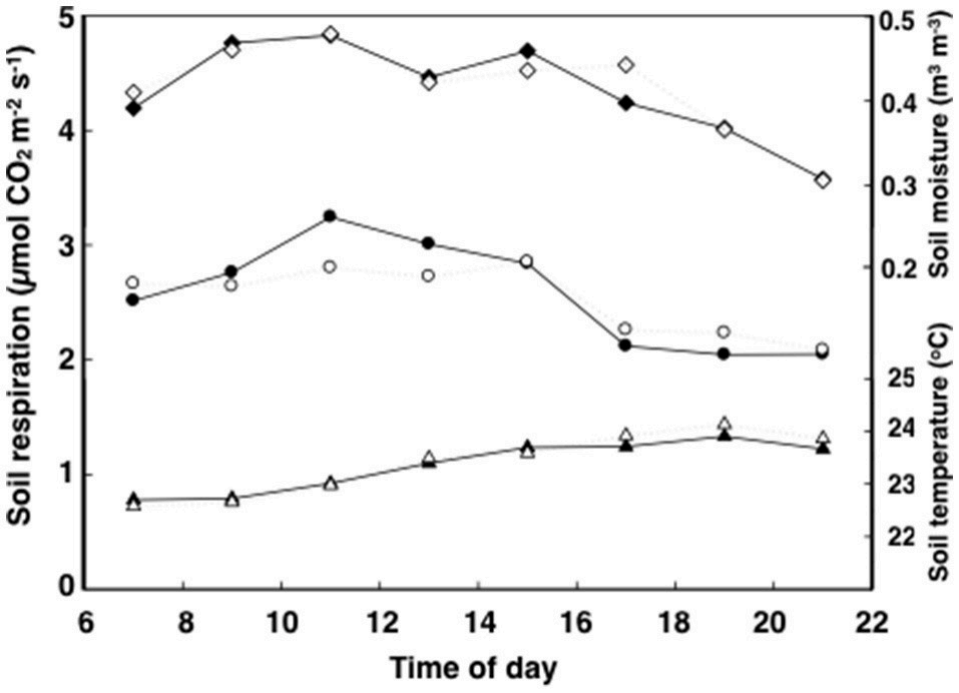
**Time course of soil respiration (circles), moisture (diamonds), and temperature (triangles) of tropical pasture plots before they were assigned to the ambient control (filled) and elevated (ambient +2°C) temperature (open) treatments**. Measurements were taken a week before treatment on 16 plots started over 3–5 days. Errors for *n* = 8 were never more than 14.6% of the mean and bars are not shown for graphical simplicity.

In general, soil respiration, soil temperature and soil moisture followed a consistent diurnal pattern (Figures 1, 2). Soil respiration was high and constant between 12:00 and 15:00 h, rapidly decreasing by 25% during the evening hours and into the night (Figure 2). In contrast, soil temperature reached a maximum value of 24°C by 18:00 to 20:00 h, slowly decreasing after that (Figure 2). Soil moisture peaked in the morning following dew water input (8:00–9:00 h) and decreased precipitously after 15:00 h (Figure 2).

**Figure 3:**
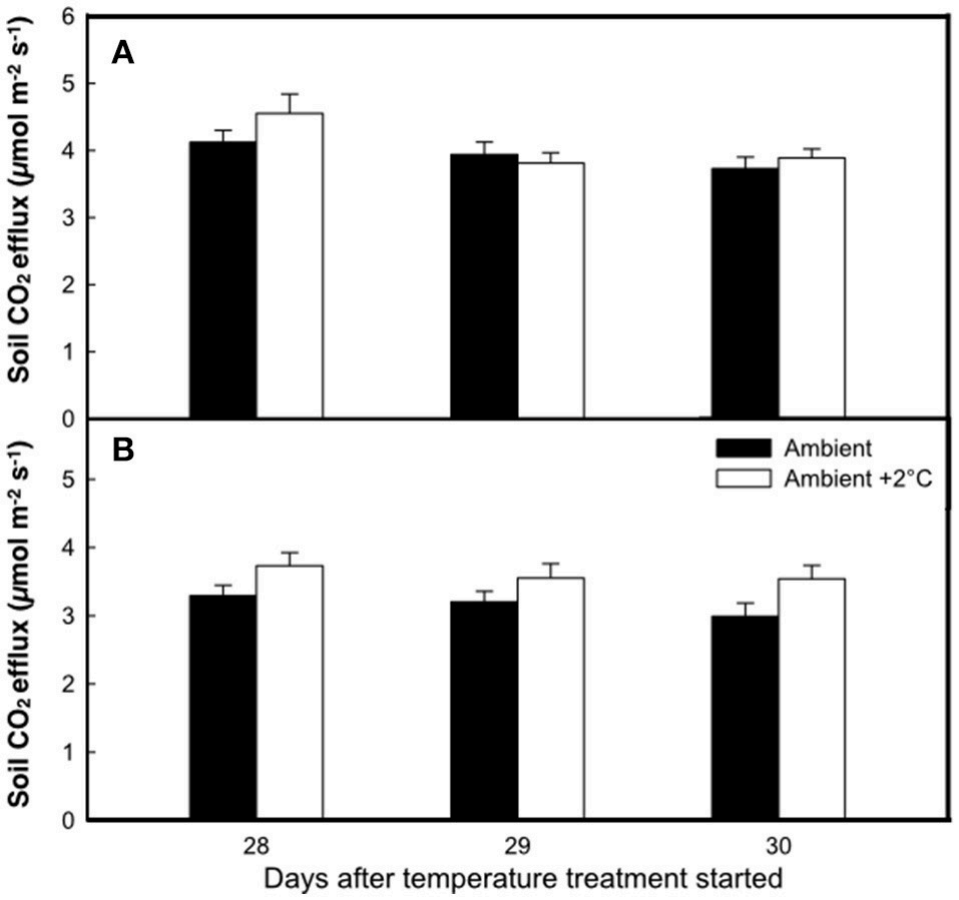
**The rate of soil respiration at the end of the growth cycle under either ambient air temperature (solid bars) or ambient +2°C temperature (white bars) measured at 12:00 h (A)** or 18:00 h **(B)**. Bars represent means of 8 plots ± SE for each of three consecutive days.

**Figure 4:**
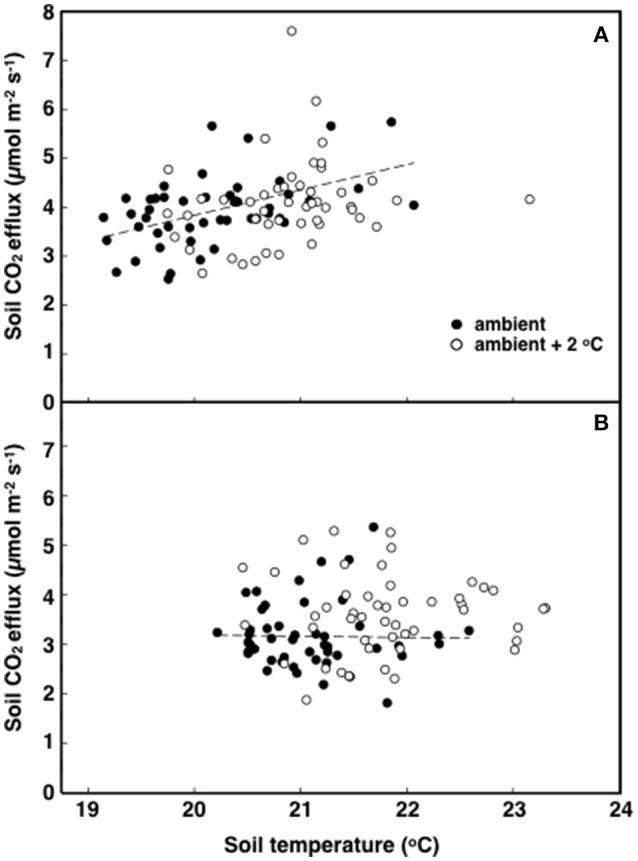
**The relationship between soil respiration and temperature in tropical pasture plots exposed to either ambient (filled) or ambient +2°C air temperature (open) in the field**. Measurements were made at 12:00 h (**A**; *p* < 0.001) or 18:00 h (**B**; *p* = 0.615).

**Figure 5:**
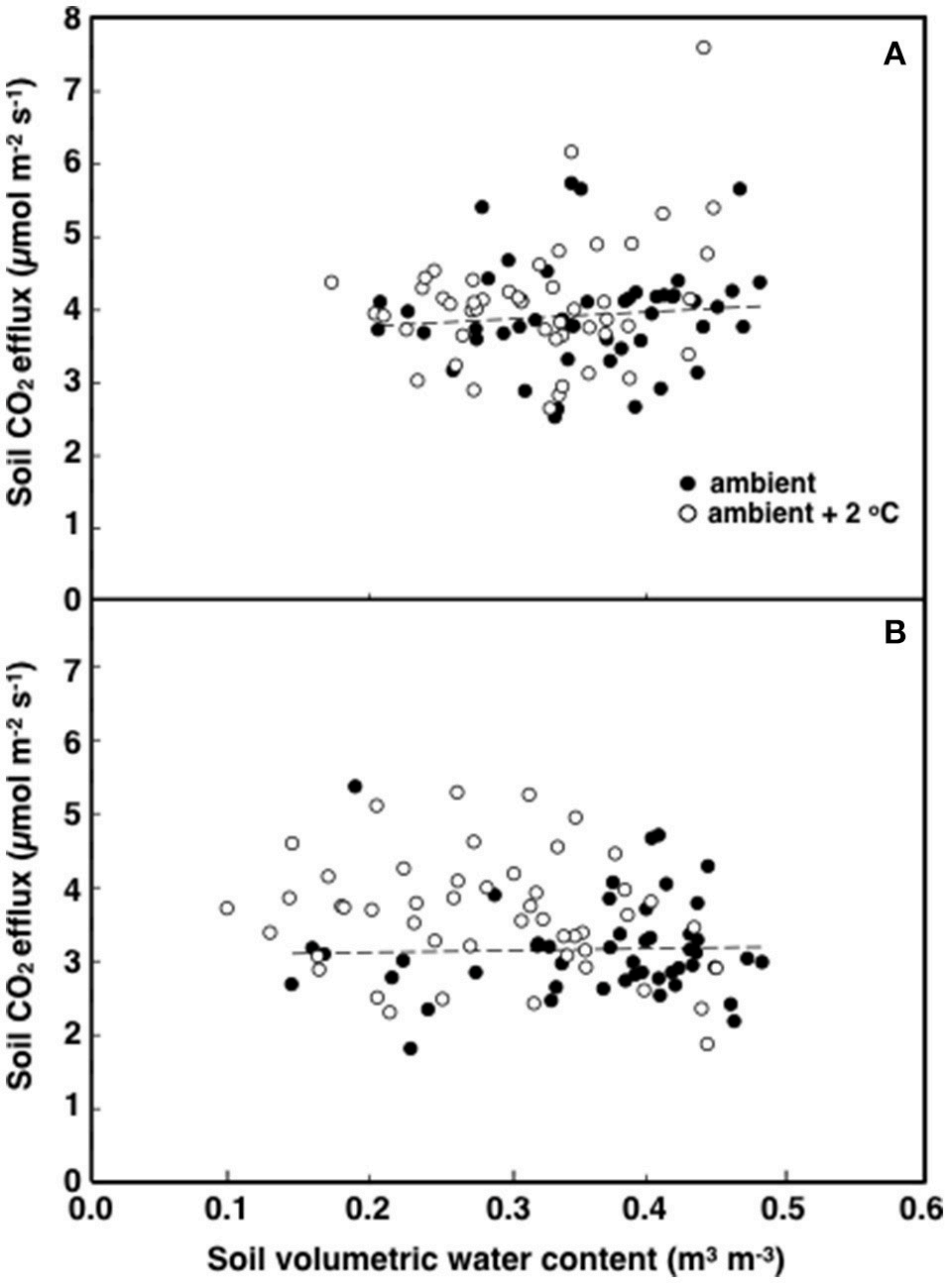
**The relationship between soil respiration and soil volumetric water content in tropical pasture plots exposed to either ambient (filled) or ambient +2°C air temperature (open) in the field**. Measurements were made at 12:00 h (**A**; *p* = 0.018) or 18:00 h (**B** graph; *p* = 0.309).

The elevated temperature treatment resulted in a significant increase (8.4%) in soil respiration when compared to ambient temperature plots (*F* = 4.28; *P* = 0.0474, Figure 3). Significant diurnal variability occurred with soil respiration 18% higher at mid-day (12:00; 4.0 μmol m^2^ s^−1^) compared to in the evening (18:00; 3.4 μmol m^2^ s^−1^) (Figure 3). Despite the 14.7% higher evening soil respiration in the warmed treatment plots relative to the ambient plots and the lack of this pattern in the mid-day (only 3.8%) the time*treatment interaction was not significant (*F* = 1.02, *P* = 0.3214; Figure 3). We also observed significant differences in soil respiration between different days progressively decreasing from day 28 to 30 (*F* = 9.98, *P* < 0.001). Although weak, soil temperature exhibited a significant positive relationship with soil respiration at 12:00 h (*R*^2^ = 0.12; *P* < 0.001) (Figure 4A), but not at 18:00 h (*P* = 0.615) (Figure 4B), the lack of an effect at 18:00 h was the reason why the covariate did not pass the even slopes model requirement to include the covariate in the grand model discussed above. Soil volumetric water content exhibited a weak and marginally significant positive relationship with soil respiration (*R*^2^ = 0.078, *P* = 0.054; Figure 5).

**Figure 6:**
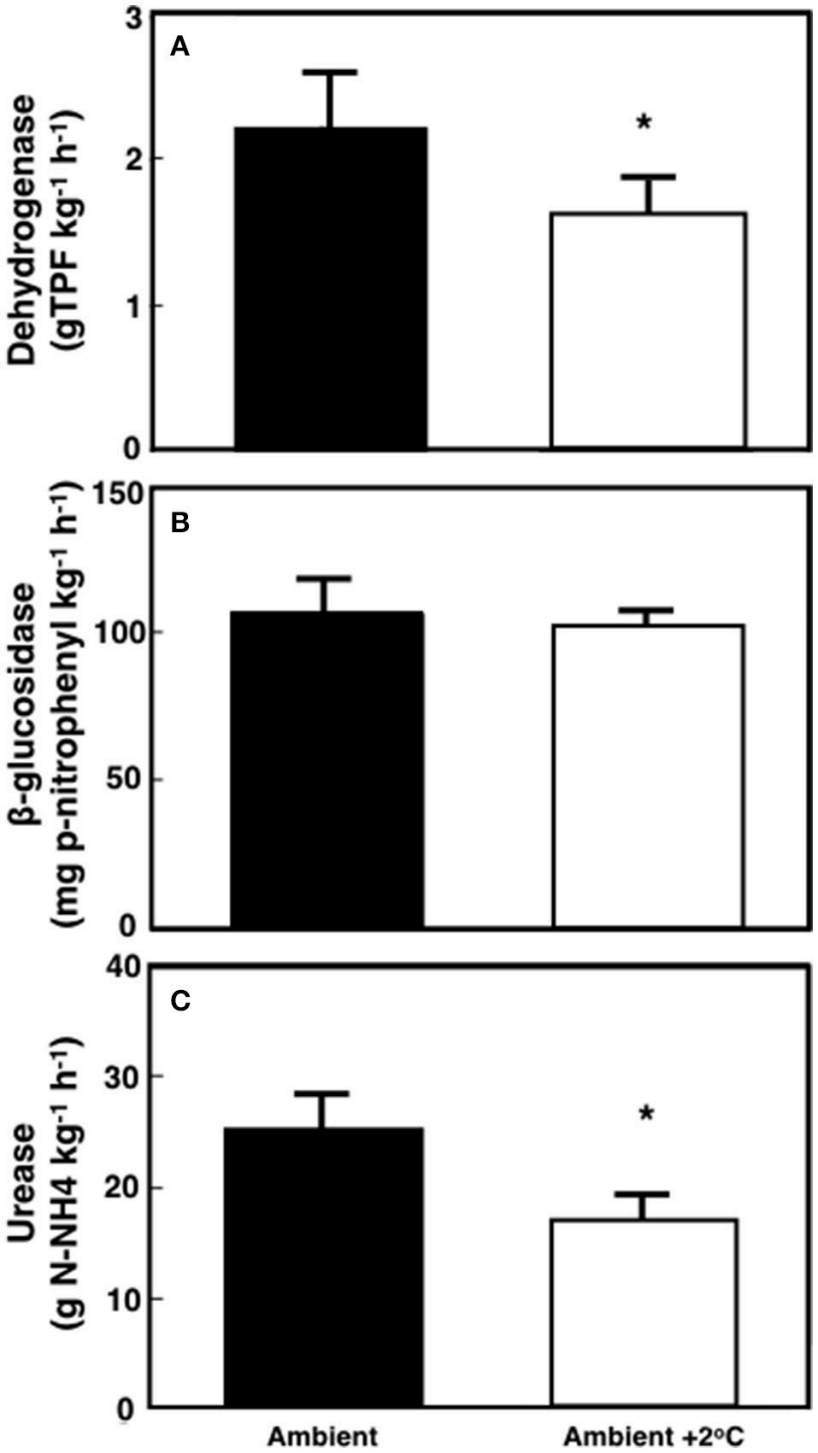
**Soil maximum enzymatic activity for dehydrogenases (TPF-trifenilformazan), β-glucosidases and ureases in tropical pasture plots grown at either ambient or at ambient +2°C air temperature in the field**. Values are averages ± SE of 8 replicates where ^*^represents statistical significant differences based on Tukey's test (*p* < 0.05).

The soil maximum enzymatic activities were measured after 29–30 days of the warming treatment (Figure 6). Soil dehydrogenase activity declined by 27% (*P* < 0.01) under warming when compared to the ambient temperature plots (Figure 6A). In contrast, the overall activity of β-glucosidase was unchanged by the temperature treatment (4.5%; *P* = 0.35 Figure 6B). The activity of soil urease decreased by 31% under +2°C air warming when compared to ambient temperature plots (*P* < 0.01; Figure 6C).

As expected, bulk soil C and N content did not change during the 30-day temperature treatment (Table 1). However, the bulk soil δ^15^N decreased at both ambient and warmed plots with respect to pretreatment days (Table 1). For ambient plots, the soil δ^15^N changed from 9.1‰ at the beginning of the experiment (T0) to 7.6‰ after 30 days of experimental warming (T30), whereas in ambient soils it changed from 8.8‰ at T0 to 7.0‰ at T30 (*P* < 0.01). The N content of *S. capitata* leaves increased during the 30-day growth period for both the ambient and the elevated temperature plots (Table 1). However, the N content of tissues increased more than 2-fold in the elevated temperature treated plants compared to the 71% increase in the ambient temperature plants (*P* < 0.01). The isotopic δ^15^N composition of leaves did not change in plants grown at ambient during the 30-day period. However, the isotopic composition of leaves from the elevated temperature plots changed from 3.0 to 2.2‰ (*P* < 0.05) at the end of the 30-day growing period. There were no changes in leaf C content or its δ^13^C value in the second harvest compared to the first harvest (30 days earlier) for the ambient or the ambient +2°C warming treatment (Table 1).

**Table 1:** **Carbon and nitrogen content in bulk soil (0–10 cm) and leaves of ***Stylosantes capitata*** with their isotopic before treatment (T0) and after 30-days of treatment (T30) of ambient temperature or ambient +2°C grown plants**.

**Time**	**T0**	**T30**	**T0**	**T30**	**T0**	**T30**	**T0**	**T30**
**Bulk Soil**	**C (gC kg**^−1^ **soil)**		δ^13^**C (‰)**		**N (gN kg**^−1^ **soil)**		δ^15^**N (‰)**	
Ambient	17.0 ± 0.5	16.6 ± 0.7	−16.3 ± 0.2	−16.4 ± 0.2	1.4 ± 0.1	1.3 ± 0.1	9.1 ± 0.2	7.6 ± 0.2^*^
+2°C	16.8 ± 0.4	16.7 ± 0.7	−16.5 ± 0.3	−16.9 ± 0.1	1.4 ± 0.1	1.3 ± 0.1	8.8 ± 0.2	7.0 ± 0.1^*^
**Leaf Tissue**	**%C**		δ^13^**C (‰)**		**%N**		δ^15^**N (‰)**	
Ambient	39.6 ± 0.3	42.5 ± 0.2	−29.0 ± 0.1	−29.0 ± 0.2	1.7 ± 0.1	2.9 ± 0.1^*^	3.3 ± 0.3	3.4 ± 0.2
+2°C	40.3 ± 0.6	42.3 ± 0.1	−29.1 ± 0.1	−29.2 ± 0.1	1.4 ± 0.1	3.2 ± 0.1^*^	3.0 ± 0.2	2.2 ± 0.3^*^^†^

*Values are averages ± SE of 8 replicates*.

* indicates a time effect and

† *indicates a treatment effect*.

After the 30-day growth period under experimental temperature treatments, aboveground dry biomass and LAI increased in both ambient and elevated air temperature treatments. At the ambient temperature, aboveground biomass increased to 361 ± 21 gm^−2^ (Table 2) after 30 days. The aboveground biomass increased to 475 ± 29 gm^−2^ at the elevated temperature treatment plots during the 30-day experimental period. Therefore, the elevated air temperature treatment stimulated aboveground biomass accumulation by 31% when compared to ambient plots (*P* < 0.01). Warming also resulted in LAI increases of 27% compared with the ambient plants (Table 2).

**Table 2:** **Leaf area index (***LAI***) and above ground dry biomass increase (AGBI) after the 30-day growth cycle of ***Stylosanthes capitate***, Vogel, grown at either ambient temperature or at ambient temperature +2°C**.

**Treatment**	***LAI* (m^2^m^−2^)**	**AGBI (g m^−2^)**
Ambient	6.8 ± 0.4	361 ± 21
Ambient + 2°C	8.7 ± 0.3^*^	475 ± 29^*^

*Values are averages ± SE of 8 replicates*.

* *Indicates statistical significant differences caused by growth temperature based on Tukey's test (p < 0.05)*.

## Discussion

Our study supports our hypotheses that soil respiration would be stimulated by the +2°C air warming treatment, although the stimulation of soil respiration was driven by a higher respiration for the +2°C air warming treatment at 18:00 h. This significant diurnal variability in soil respiration (higher during the day compared to that of the evening) underpins the relevance of plant photosynthetic activity on soil respiration rates (Hopkins et al., 2013). Additionally, the positive but weak relationship between soil temperature and soil respiration at 12:00 h was similar in both ambient and the elevated temperature treatment, indicating a similar physiological soil response to warming. Soil moisture and temperature did not appear to accelerate N and C cycling since maximum potential activities of soil enzymes decreased under air and soil warming (Figure 6).

Soil temperature and moisture are the factors most commonly related to temporal variation in CO_2_ efflux from soils (Hopkins et al., 2013). Low soil moisture can reduce CO_2_ efflux from soils while soil warming generally intensifies soil respiration (Davidson and Janssens, 2006; Mikkelsen et al., 2007; Yang et al., 2012; Lu et al., 2013), perhaps by increasing the activity of decomposers (Hopkins et al., 2013; Cheng et al., 2014). In arid regions, a temperature-soil moisture interaction term explains most of the diurnal and seasonal variations in CO_2_ efflux (Wildung et al., 1975; Davidson et al., 2000). In temperate grasslands, however, moisture and photosynthesis have a bigger influence on soil respiration than soil temperature, at least at seasonal time scales (Gomez-Casanovas et al., 2012). These variations in ecosystem and climate controls over soil respiration may be due to the different sensitivities to soil factors by the autotrophic and heterotrophic components of soil respiration (Taneva and Gonzalez-Meler, 2011; Hopkins et al., 2013). In this study, an indicator of these different sensitivities of soil respiration to soil temperature is a modest but significant response of soil respiration to elevated air and soil temperature. The low correlation of soil respiration to temperature in this Neotropical grassland suggests a thermal acclimation of plants and soil microorganisms as seen elsewhere (Luo et al., 2001; Wood et al., 2012) and the role of plant activity on modulating belowground C flux (Gomez-Casanovas et al., 2012; Hopkins et al., 2013), suggesting a dominance of soil respiration by root respiration at 18:00 h (Figures 2, 4, and 5).

In other tropical pastures in Amazonia, seasonal variations in soil respiration were explained by changes in volumetric soil water content rather than temperature (Gaumont-Guay et al., 2006; Tian et al., 2011; Atarashi-Andoh et al., 2012; Zhao et al., 2013). Recent findings from temperate forests suggest that soil temperature enhances belowground processes by increasing the respiration of roots, microbes or both (Epron et al., 2001; Caquet et al., 2012). Some of the observed increases in soil respiration in response to warming may have been caused by increases in belowground C allocation by plants (Reich et al., 2006; Hopkins et al., 2013; Yin et al., 2013; Cheng et al., 2014; Gonzalez-Meler et al., 2014). Presumably, soil respiration enhancement under the elevated temperature treatment seen in this study was due to enhanced plant activity and by the lack of increase in the activity of major soil enzymes (Figure 3). These results are consistent with the notion of enhanced root respiration at the elevated temperature treatment when compared to ambient plots.

In our study, soil respiration increased under air warming treatment despite moderate soil warming (~0.8°C). Soil respiration was also correlated with changes in N dynamics caused by a 30-day air temperature increase in this tropical pasture. Temperature may have also affected processes, such as N fixation or N mineralization via nitrification and denitrification as described elsewhere (Sierra, 2002; Houlton et al., 2008; Bai et al., 2013; Kuster et al., 2013). In this 30-day experimental warming experiment, N cycling was affected mainly by decreasing soil urease activity over ambient soils (Figure 6). Leaf N content was higher under elevated than under ambient temperature conditions (Table 1) and the leaf N isotopic composition was stable during the 30-day period at the ambient treatment but became depleted in leaves of plants exposed to the +2°C air warming treatment (Table 1). These results (increased N content and depleted ^15^N composition) suggest a higher proportion of leaf N coming from soil processes, such as N_2_- fixation, as N_2_-fixation is known to increase under conditions of higher N demand by legumes (Salvagiotti et al., 2008). Furthermore, δ^15^N values may have a direct correlation with N_2_-fixation (Belnap, 2001). Although N-fixation was not specifically measured, the reduced soil urease enzymatic potential (Figure 6), a proxy for soil N mineralization, and the depletion of leaf ^15^N are both consistent with reduced proportion of plant N demand from soils relative to N provided by N-fixation.

Long-term experimental warming has resulted in higher soil C and N concentrations in some cases (Caprez et al., 2012) but not in others (Lu et al., 2013). Our experiments showed a decrease in C oxidation and N harvesting enzymes (Figure 6) rather than an increase. The slightly lower water content in soils from warmed plots (*P* < 0.001; Figure 1) may have contributed to this effect, although plants did not show symptoms of water stress (Table 1). An increase in air and soil temperature both caused a reduction in microbial decomposition *via* indirect effects of temperature on soil water availability in other warming studies in temperate systems (Davidson and Janssens, 2006; Belay-Tedla et al., 2009; Liu et al., 2009a; Wood et al., 2012). Other warming studies have described an initial increase in microbial activity due to warming-induced increase in respiration, followed by a rapid return to initial rates due to biochemical acclimation, substrate limitations, or after shifts in microbial community composition (Allison et al., 2010). The effects of soil moisture and temperature on soil processes might be different depending on the specificity of soil enzymes, as some enzymes are more sensitive to temperature or moisture than others (Sardans et al., 2008). Warming-induced soil drying often reduces microbial biomass and microbial activity, and changes microbial community composition (Ainsworth et al., 2003; Liu et al., 2009b; Kuzyakov and Gavrichkova, 2010; Schindlbacher et al., 2011; Vanhala et al., 2011; Fu et al., 2012). However, our results are not consistent with observations from non-tropical systems. The effects of a 30-day experimental air warming do not seem to increase the activity of decomposers, potentially reducing the positive feedback of increased temperature on decomposition rates of soil C in tropical pastures.

It is possible that modest decreases in soil moisture could lead to a decrease in soil N mineralization potential (Bai et al., 2013), preventing an increase in enzyme capacity at the elevated temperature treatment (Figure 6). Houlton et al. (2008) found that N inputs from N fixation increase with soil temperature but plateau or begin to decline at temperatures above 26°C. This could result in lower N fixation rates at the elevated temperature treatment. However, the leaf ^15^N data suggest such temperature-induce decline of N-fixation, if any, did not negatively affect leaf N content or the proportion of N uptake from N-fixation (Table 1). The elevated air temperature treatment may have increased N demand by accelerating plant growth (Saxe et al., 2002; Way and Oren, 2010). Aboveground biomass and leaf area index was indeed higher in plants grown at the elevated temperature treatment when compared to ambient ones (Table 2). Therefore, increases in plant biomass is likely to enhance root activity and N-fixation causing the increase seen in soil respiration during the evening and early night hours (Figure 4; Ryan and Law, 2005).

In conclusion, increased air (and soil) temperature by 2°C did not result in a consistent increase in soil respiration at the end of the 30-day warming experiment. Based on plant tissue values of ^13^C, a moderate decrease in soil water content did not appear to cause plant water stress in the warmed plots. However, warming and perhaps minor changes in soil moisture affected the microbial activity responsible for soil C oxidation and N harvesting. Apparent increases in N-fixation may have increased leaf N content and fueled aboveground plant growth. As a result, soil respiration appeared to be dominated by plant root activity and not by the activity of microbial decomposers. Rapid shifts in plant and soil N dynamics in response to the first 30-day +2°C warming period field experiment made in the Southern Hemisphere was surprising and unlike results from temperate grasslands and other ecosystems. Our results highlight the need for experimental climate change manipulations in tropical ecosystems to develop key response variables of tropical agriculture to climate change.

## Author contributions

MG-M, have written the drafts and coordinated data analysis. LS, data sampling and figures elaboration. ED, data analysis and collaboration on drafting. CF, Data analysis and collaboration on drafting. CM, Experimental management, figures and drafting.

### Conflict of interest statement

The authors declare that the research was conducted in the absence of any commercial or financial relationships that could be construed as a potential conflict of interest.
